# 

**Published:** 1889-03-09

**Authors:** 


PERMANENTLY ENLARGED TO 32 PAGES.

PRICE ONE PENNY.

No. 128, Vol. V.—March 9th, 1889.

[Registered at the General Post Office as a Newspaper.]

Per Annum, 6s. 6d., post free.
Monthly Parts. 6d.; post free, 7½d.
Ditto per Ann. 7s. 6d., post free.

### THE HOSPITAL

A WEEKLY INSTITUTIONAL JOURNAL OF

